# Supplementary material for: Basal Forebrain Volume is Associated With Cortical Amyloid Burden in Cognitively Unimpaired Older Adults at Varying Genetic Risk for Alzheimer’s Disease
Source: Am J Geriatr Psychiatry Open Sci Educ Pract. Author manuscript; Available in PMC 2026 Oct 17. (PMC13674689; doi:10.1016/j.osep.2026.04.002)
Supplement: Supplementary file 1 [file NIHMS2213082-supplement-1.docx]

Basal forebrain volume is associated with cortical amyloid burden in cognitively unimpaired older adults at varying genetic risk for Alzheimer’s disease

Gawon Cho, Ph.D. ^1^; Kaitlin Kelly^2,3^; Takuya Toyonaga, M.D., Ph.D.^4^; Brienne Miner, M.D., M.HS.^1^; Joel Gelernter, M.D.^5-8^; Pradeep Varma, M.D.^4^; Christopher H. van Dyck, M.D. ^2,5,7,9^; Adam P. Mecca, M.D., Ph.D.^2,5^; Ryan O’Dell, M.D., Ph.D.^2,5*^

^1^ Department of Internal Medicine, Yale School of Medicine, New Haven, CT, USA

^2^ Yale Alzheimer’s Disease Research Unit, Yale School of Medicine, New Haven, CT, USA

^3^ Geisinger Commonwealth School of Medicine, Scranton, PA, USA

^4^ Department of Radiology and Biomedical Imaging, Yale University School of Medicine, New Haven, CT, USA

^5^ Department of Psychiatry, Yale School of Medicine, New Haven, CT, USA

^6^ Veterans Affairs Connecticut Healthcare System, West Haven, CT, USA

^7^ Department of Neuroscience, Yale University School of Medicine, New Haven, CT, USA

^8^ Department of Genetics, Yale University School of Medicine, New Haven, CT, USA

^9^ Department of Neurology, Yale University School of Medicine, New Haven, CT, USA

*Corresponding Author:

Ryan S. O’Dell, M.D., Ph.D.

Alzheimer’s Disease Research Unit

Yale University School of Medicine

One Church Street, 8^th^ Floor

New Haven, CT 06510

tel +1 203 764-8100

fax +1 203 764-8111

Email: [ryan.odell@yale.edu](mailto:ryan.odell@yale.edu)

**Table of Contents:**

| **Content** | **Pages** |
| --- | --- |
| Supplementary Table 1. Participant characteristics stratified by Aβ positivity using a mean global cortical [^11^C]PiB *DVR* threshold of 1.2 | 3 |
| Supplementary Table 2. Participant characteristics stratified by Aβ positivity using a mean global cortical [^11^C]PiB *DVR* threshold of 1.08 | 4 |
| Supplementary Table 3. Sex differences in the relationship between basal forebrain substructural volumes and global amyloid burden | 5 |
| Supplementary Table 4. Relationship between basal forebrain substructural volumes and regional amyloid burden | 6 |
| Supplementary Table 5. Association between Freesurfer region volumes and global amyloid burden without partial volume correction | 7-8 |
| Supplementary Table 6. Association between Freesurfer region volumes and global amyloid burden with partial volume correction using Iterative Yang method | 9-10 |
| Supplementary Table 7. Relationship between basal forebrain and cortical volume across all FreeSurfer regions | 11-12 |
| Supplementary Figure 1. Mean global cortical amyloid burden by *APOE* genotype with (B) and without (A) partial volume correction | 13 |
| Supplementary Figure 2. Amyloid burden in basal forebrain substructures by *APOE* genotype after partial volume correction | 14 |
| Supplementary Figure 3. Association between partial volume-corrected untransformed and log-transformed global cortical amyloid burden and (A) Ch1-3 volume and () Ch4 volume | 15 |
| Supplementary Figure 4. Association between untransformed and log-transformed global cortical amyloid burden and (A) Ch1-3 volume and (B) Ch4 volume in Aβ + and Aβ- participants using a [^11^C]PiB *DVR* threshold of 1.2 | 16 |
| Supplementary Figure 5. Group differences in BFCS volume by amyloid status using a mean global cortical amyloid *DVR* threshold of 1.2 | 17 |
| Supplementary Figure 6. Association between untransformed and log-transformed global cortical amyloid burden and (A) Ch1-3 volume and (B) Ch4 volume in Aβ + and Aβ- participants using a [^11^C]PiB *DVR* threshold of 1.08 | 18 |
| Supplementary Figure 7. Association between partial volume-corrected untransformed and log-transformed global cortical amyloid burden and (A) hippocampal, (B) parahippocampal, (C) entorhinal, and (D) amygdala volume | 19 |
| Supplementary Figure 8. Association between untransformed and log-transformed global cortical amyloid burden and (A) hippocampal, (B) parahippocampal, (C) entorhinal, and (D) amygdalar volume in Aβ + and Aβ- participants using a [^11^C]PiB *DVR* threshold of 1.2 | 20 |
| Supplementary Figure 9. Group differences in medial temporal lobe volume by amyloid status using a mean global cortical amyloid *DVR* threshold of 1.2 | 21 |
| Supplementary Figure 10. Association between untransformed and log-transformed global cortical amyloid burden and (A) hippocampal, (B) parahippocampal, (C) entorhinal, and (D) amygdala volume in Aβ+ and Aβ- participants using a [^11^C]PiB *DVR* threshold of 1.08 | 22 |

**Supplementary Table 1.** Participant characteristics stratified by Aβ positivity using a mean global cortical [^11^C]PiB *DVR* threshold of 1.2

|  | **Amyloid Negative** | **Amyloid Positive** | **t/χ^2^** | ***P*** |
| --- | --- | --- | --- | --- |
| **Participants (n)** | 34 | 11 |  |  |
| **Sex (M/F)** | 16/18 | 5/6 | 0.009^a^ | 0.93 |
| **Age (years)** | 58.2 (4.6, 50.1-65.9) | 61.9 (4.1, 53.7-66.9) | 2.39^b^ | 0.02* |
| **Education (years)** | 16.2 (1.8, 12-20) | 16.8 (2.0, 12-18) | 1.00^b^ | 0.33 |
| **Global Amyloid (*DVR*)** | 1.0 (0.1, 0.9-1.2) | 1.4 (0.2, 1.2-1.8) | 8.21^b^ | < 0.0001* |
| ***APOE* Genotype** |  |  | 6.74^c^ | 0.03* |
| **ɛ3ɛ3** | 14 | 1 |  |  |
| **ɛ3ɛ4** | 12 | 3 |  |  |
| **ɛ4ɛ4** | 8 | 7 |  |  |

Data for continuous variables are mean (SD, range). *P* values are for t-tests (continuous variables) or *χ*^2^ tests (categorical variables). * *P* < 0.05. ^a^Degrees of freedom: χ^2^(1). ^b^Degrees of freedom: t(43). ^c^Degrees of freedom: χ^2^(2). Abbreviations: *Aβ*, amyloid beta; *PiB*; Pittsburg compound B; *DVR*, distribution volume ratio; *APOE*, Apolipoprotein E.

**Supplementary Table 2.** Participant characteristics stratified by Aβ positivity using a mean global cortical [^11^C]PiB *DVR* threshold of 1.08

Data for continuous variables are mean (SD, range). *P* values are for t-tests (continuous variables) or *χ*^2^ tests (categorical variables). * *P* < 0.05. ^a^Degrees of freedom: χ^2^(1). ^b^Degrees of freedom: t(43). ^c^ Degrees of freedom: χ^2^(2). Abbreviations: *Aβ*, amyloid beta; *PiB*; Pittsburg compound B; *DVR*, distribution volume ratio; *APOE*, apolipoprotein E.

|  | **Amyloid Negative** | **Amyloid Positive** | **t/χ^2^** | ***P*** |
| --- | --- | --- | --- | --- |
| **Participants (n)** | 24 | 21 |  |  |
| **Sex (M/F)** | 9/15 | 12/9 | 1.74^a^ | 0.19 |
| **Age (years)** | 57.5 (4.4, 50.1-64.8) | 61.0 (4.4, 51.0-66.9) | 2.39^b^ | 0.02* |
| **Education (years)** | 16.1 (1.7, 12-18) | 16.6 (2.0, 12-20) | 0.96 ^b^ | 0.34 |
| **Global Amyloid (*DVR*)** | 1.0 (0.05, 0.9-1.1) | 1.3 (0.19, 1.1-1.8) | 7.30^b^ | < 0.001* |
| **APOE ɛ4 Copy Number** |  |  | 13.39^c^ | 0.001* |
| **0 copies** | 13 | 2 |  |  |
| **1 copy** | 8 | 7 |  |  |
| **2 copies** | 3 | 12 |  |  |

**Supplementary Table 3.** Sex differences in the relationship between basal forebrain substructural volumes and global amyloid burden

|  | **Global cortical amyloid burden (*DVR*) by basal forebrain region** | |
| --- | --- | --- |
|  | **Ch1-3, bilateral** | **Ch4, bilateral** |
| **Women** | Pearson *r* = -0.20, *P* = 0.17 (No transform)  Pearson *r* = -0.29, *P* = 0.08 (Log-transformed amyloid) | Pearson *r* = -0.66, *P* = 0.0002 (No transform)  Pearson *r* = -0.57, *P* = 0.002 (Log-transformed amyloid) |
| **Men** | Pearson *r* = 0.03, *P* = 0.56 (No transform)  Pearson *r* = 0.09, *P* = 0.66 (Log-transformed amyloid) | Pearson *r* = 0.21, *P* = 0.82 (No transform)  Pearson *r* = 0.17, *P* = 0.76 (Log-transformed amyloid) |
| **Sex differences** | Δr_no transform_ = 0.24, *P* = 0.40 | Δr_no transform_ = 0.87, *P* = 0.002 |

Degrees of freedom for Pearson correlation coefficients with untransformed amyloid burden is 22 for women and 19 for men.

**Supplementary Table 4.** Relationship between basal forebrain substructural volumes and regional amyloid burden

| **Method of regional amyloid burden quantification** | **Basal forebrain substructures** | | | | |
| --- | --- | --- | --- | --- | --- |
|  | **Ch1-3, Bilateral** | |  | **Ch4, Bilateral** | |
|  | ***r*** | ***P*** |  | ***r*** | ***P*** |
| **Regional amyloid burden without PVC** | 0.25 | 0.95 |  | -0.07 | 0.33 |
| **Regional amyloid burden after IY-PVC** | 0.30 | 0.98 |  | -0.12 | 0.21 |

*P*-values are derived from one-sided tests based on the assumption of a negative correlation. Degrees of freedom for Pearson correlation coefficients is 43. Abbreviations. *Ch*, Cholinergic nuclei; *IY-PVC*, Iterative Yang partial volume correction; r = Pearson r.

**Supplementary Table 5.** Association between Freesurfer region volumes and global amyloid burden without partial volume correction

| **Bilateral region** | **Full sample** | | **Amyloid positive** | | **Amyloid negative** | |
| --- | --- | --- | --- | --- | --- | --- |
|  | **Pearson *r*** | ***P*** | **Pearson *r*** | ***P*** | **Pearson *r*** | ***P*** |
| bl_frontalpole | -0.29 | 0.06 | -0.09 | 0.78 | -0.29 | 0.09 |
| bl_superiorfrontal | -0.21 | 0.18 | 0.30 | 0.37 | 0.16 | 0.37 |
| bl_rostralmiddlefrontal | -0.21 | 0.17 | -0.11 | 0.74 | 0.02 | 0.93 |
| bl_caudalmiddlefrontal | -0.13 | 0.39 | 0.08 | 0.81 | 0.09 | 0.63 |
| bl_parsorbitalis | -0.26 | 0.08 | -0.36 | 0.28 | -0.20 | 0.27 |
| bl_parsopercularis | -0.12 | 0.43 | 0.35 | 0.29 | 0.13 | 0.45 |
| bl_parstriangularis | 0.10 | 0.53 | 0.12 | 0.73 | 0.16 | 0.35 |
| bl_lateralorbitofrontal | -0.35* | 0.02 | -0.49 | 0.12 | -0.28 | 0.11 |
| bl_medialorbitofrontal | -0.27 | 0.08 | -0.28 | 0.41 | -0.35* | 0.05 |
| bl_temporalpole | -0.35* | 0.02 | -0.57 | 0.07 | -0.13 | 0.46 |
| bl_entorhinal | -0.05 | 0.73 | -0.49 | 0.13 | -0.03 | 0.85 |
| bl_parahippocampal | -0.26 | 0.08 | -0.31 | 0.36 | -0.11 | 0.54 |
| bl_Hippocampus | -0.15 | 0.33 | 0.58 | 0.06 | -0.15 | 0.40 |
| bl_Amygdala | -0.02 | 0.91 | 0.31 | 0.35 | -0.13 | 0.47 |
| bl_inferiortemporal | 0.25 | 0.10 | 0.41 | 0.21 | -0.02 | 0.91 |
| bl_fusiform | -0.18 | 0.23 | -0.43 | 0.19 | -0.03 | 0.88 |
| bl_middletemporal | -0.49* | 0.0006 | -0.36 | 0.28 | -0.24 | 0.18 |
| bl_bankssts | -0.32* | 0.03 | 0.06 | 0.87 | -0.19 | 0.28 |
| bl_superiortemporal | -0.34* | 0.02 | -0.33 | 0.32 | -0.23 | 0.18 |
| bl_transversetemporal | 0.09 | 0.55 | 0.27 | 0.43 | -0.04 | 0.82 |
| bl_supramarginal | -0.07 | 0.66 | 0.12 | 0.73 | 0.10 | 0.56 |
| bl_insula | -0.33* | 0.03 | -0.55 | 0.08 | -0.10 | 0.57 |
| bl_rostralanteriorcingulate | -0.35* | 0.02 | -0.53 | 0.09 | -0.0005 | 1.00 |
| bl_caudalanteriorcingulate | -0.33* | 0.03 | -0.31 | 0.36 | -0.42* | 0.01 |
| bl_posteriorcingulate | -0.32* | 0.03 | -0.34 | 0.31 | -0.02 | 0.91 |
| bl_isthmuscingulate | -0.27 | 0.07 | -0.01 | 0.99 | -0.08 | 0.63 |
| bl_precuneus | -0.03 | 0.84 | 0.04 | 0.90 | 0.01 | 0.96 |
| bl_paracentral | -0.26 | 0.09 | 0.20 | 0.56 | -0.18 | 0.31 |
| bl_postcentral | 0.06 | 0.68 | 0.81* | 0.003 | 0.14 | 0.43 |
| bl_precentral | -0.02 | 0.90 | 0.28 | 0.41 | -0.10 | 0.58 |
| bl_superiorparietal | 0.07 | 0.66 | 0.11 | 0.75 | 0.04 | 0.80 |
| bl_inferiorparietal | -0.28 | 0.07 | 0.33 | 0.33 | -0.35* | 0.04 |
| bl_lateraloccipital | -0.27 | 0.07 | -0.09 | 0.80 | -0.18 | 0.31 |
| bl_cuneus | -0.28 | 0.06 | -0.41 | 0.22 | -0.22 | 0.21 |
| bl_pericalcarine | -0.13 | 0.41 | -0.08 | 0.81 | -0.18 | 0.30 |
| bl_lingual | -0.29 | 0.05 | -0.19 | 0.58 | -0.19 | 0.28 |
| bl_Thalamus_Proper | -0.36* | 0.02 | 0.08 | 0.82 | -0.44 | 0.01 |
| bl_Caudate | -0.05 | 0.76 | 0.10 | 0.77 | -0.10 | 0.59 |
| bl_Putamen | -0.24 | 0.12 | 0.12 | 0.73 | -0.32 | 0.07 |
| bl_Pallidum | -0.20 | 0.18 | 0.05 | 0.89 | -0.45* | 0.01 |
| bl_Accumbens_area | -0.24 | 0.12 | -0.01 | 0.97 | -0.18 | 0.30 |
| bl_VentralDC | -0.24 | 0.12 | -0.11 | 0.76 | -0.42* | 0.01 |

Degrees of freedom for Pearson correlation coefficients is 43.

**Supplementary Table 6.** Association between Freesurfer region volumes and global amyloid burden with partial volume correction using Iterative Yang method

| **Bilateral region** | **Full sample** | | **Amyloid positive** | | **Amyloid negative** | |
| --- | --- | --- | --- | --- | --- | --- |
|  | **Pearson *r*** | ***P*** | **Pearson *r*** | ***P*** | **Pearson *r*** | ***P*** |
| bl_frontalpole | -0.34* | 0.02 | -0.18 | 0.40 | -0.41 | 0.07 |
| bl_superiorfrontal | -0.26 | 0.08 | -0.35 | 0.09 | -0.47* | 0.03 |
| bl_rostralmiddlefrontal | -0.23 | 0.13 | -0.33 | 0.11 | -0.50* | 0.02 |
| bl_caudalmiddlefrontal | -0.20 | 0.18 | -0.32 | 0.12 | -0.16 | 0.48 |
| bl_parsorbitalis | -0.26 | 0.09 | -0.16 | 0.46 | -0.26 | 0.25 |
| bl_parsopercularis | -0.12 | 0.42 | -0.16 | 0.46 | -0.15 | 0.51 |
| bl_parstriangularis | 0.10 | 0.53 | -0.01 | 0.98 | -0.32 | 0.16 |
| bl_lateralorbitofrontal | -0.32* | 0.03 | -0.20 | 0.34 | -0.52* | 0.02 |
| bl_medialorbitofrontal | -0.29 | 0.05 | -0.12 | 0.59 | -0.21 | 0.35 |
| bl_temporalpole | -0.34* | 0.02 | -0.32 | 0.13 | -0.20 | 0.38 |
| bl_entorhinal | -0.03 | 0.85 | -0.26 | 0.23 | -0.33 | 0.15 |
| bl_parahippocampal | -0.23 | 0.13 | -0.15 | 0.50 | -0.22 | 0.33 |
| bl_Hippocampus | -0.16 | 0.30 | 0.10 | 0.65 | -0.32 | 0.15 |
| bl_Amygdala | 0.02 | 0.91 | 0.15 | 0.49 | -0.33 | 0.14 |
| bl_inferiortemporal | 0.24 | 0.12 | 0.21 | 0.31 | -0.18 | 0.43 |
| bl_fusiform | -0.21 | 0.17 | -0.17 | 0.43 | -0.05 | 0.83 |
| bl_middletemporal | -0.49* | 0.0006 | -0.49* | 0.02 | -0.13 | 0.59 |
| bl_bankssts | -0.35* | 0.02 | -0.27 | 0.20 | -0.18 | 0.43 |
| bl_superiortemporal | -0.32* | 0.03 | -0.27 | 0.21 | -0.35 | 0.12 |
| bl_transversetemporal | 0.13 | 0.40 | 0.23 | 0.28 | -0.25 | 0.27 |
| bl_supramarginal | -0.09 | 0.55 | -0.17 | 0.41 | -0.35 | 0.12 |
| bl_insula | -0.35* | 0.02 | -0.55* | 0.005 | -0.04 | 0.87 |
| bl_rostralanteriorcingulate | -0.37* | 0.01 | -0.52 | 0.01 | -0.24 | 0.29 |
| bl_caudalanteriorcingulate | -0.34* | 0.02 | -0.17 | 0.42 | -0.46* | 0.03 |
| bl_posteriorcingulate | -0.32* | 0.03 | -0.28 | 0.18 | -0.19 | 0.40 |
| bl_isthmuscingulate | -0.27 | 0.08 | -0.25 | 0.25 | 0.02 | 0.92 |
| bl_precuneus | -0.07 | 0.63 | -0.16 | 0.46 | -0.15 | 0.53 |
| bl_paracentral | -0.27 | 0.07 | -0.10 | 0.64 | -0.41 | 0.07 |
| bl_postcentral | 0.03 | 0.84 | 0.13 | 0.53 | -0.08 | 0.72 |
| bl_precentral | -0.07 | 0.64 | 0.03 | 0.90 | 0.01 | 0.96 |
| bl_superiorparietal | 0.04 | 0.77 | 0.06 | 0.79 | -0.17 | 0.45 |
| bl_inferiorparietal | -0.29 | 0.05 | -0.10 | 0.65 | -0.13 | 0.58 |
| bl_lateraloccipital | -0.25 | 0.09 | -0.15 | 0.49 | -0.13 | 0.58 |
| bl_cuneus | -0.30* | 0.04 | -0.31 | 0.14 | 0.15 | 0.52 |
| bl_pericalcarine | -0.15 | 0.34 | -0.07 | 0.76 | 0.25 | 0.27 |
| bl_lingual | -0.26 | 0.08 | -0.16 | 0.45 | 0.42 | 0.06 |
| bl_Thalamus_Proper | -0.37* | 0.01 | -0.23 | 0.27 | -0.58 | 0.01 |
| bl_Caudate | 0.004 | 0.98 | 0.21 | 0.31 | -0.04 | 0.86 |
| bl_Putamen | -0.18 | 0.23 | 0.08 | 0.70 | -0.21 | 0.37 |
| bl_Pallidum | -0.19 | 0.20 | 0.13 | 0.54 | -0.09 | 0.68 |
| bl_Accumbens_area | -0.20 | 0.18 | -0.08 | 0.71 | -0.37 | 0.10 |
| bl_VentralDC | -0.22 | 0.15 | 0.05 | 0.82 | -0.24 | 0.30 |

Degrees of freedom for Pearson correlation coefficients is 43.

| **Supplementary Table 7.** Relationship between basal forebrain and cortical volume across all FreeSurfer regions | | | | | |  |
| --- | --- | --- | --- | --- | --- | --- |
| **Left Hemisphere Region** | **Ch1-3**  **Pearson *r* (*P*)** | **Ch4**  **Pearson *r* (*P*)** | **Right Hemisphere Region** | **Ch1-3**  **Pearson *r* (*P*)** | **Ch4**  **Pearson *r* (*P*)** | |
| ctx-lh-frontalpole | -0.08 (0.59) | 0.32 (0.03*) | ctx-rh-frontalpole | 0.16 (0.29) | 0.19 (0.21) | |
| ctx-lh-superiorfrontal | 0.37 (0.01*) | 0.16 (0.28) | ctx-rh-superiorfrontal | 0.25 (0.10) | 0.13 (0.40) | |
| ctx-lh-rostralmiddlefrontal | 0.26 (0.08) | 0.14 (0.36) | ctx-rh-rostralmiddlefrontal | 0.24 (0.11) | 0.11 (0.49) | |
| ctx-lh-caudalmiddlefrontal | 0.21 (0.17) | 0.06 (0.69) | ctx-rh-caudalmiddlefrontal | 0.28 (0.07) | 0.04 (0.78) | |
| ctx-lh-parsorbitalis | -0.01 (0.98) | 0.33 (0.03*) | ctx-rh-parsorbitalis | 0.06 (0.69) | 0.30 (0.05*) | |
| ctx-lh-parsopercularis | 0.04 (0.79) | -0.16 (0.30) | ctx-rh-parsopercularis | 0.04 (0.81) | 0.05 (0.72) | |
| ctx-lh-parstriangularis | 0.15 (0.32) | -0.16 (0.29) | ctx-rh-parstriangularis | -0.15 (0.33) | -0.16 (0.29) | |
| ctx-lh-lateralorbitofrontal | 0.22 (0.14) | 0.31 (0.04*) | ctx-rh-lateralorbitofrontal | 0.46 (0.001*) | 0.27 (0.07) | |
| ctx-lh-medialorbitofrontal | -0.22 (0.15) | 0.11 (0.48) | ctx-rh-medialorbitofrontal | 0.18 (0.25) | 0.06 (0.70) | |
| ctx-lh-temporalpole | -0.22 (0.15) | 0.33 (0.03*) | ctx-rh-temporalpole | -0.10 (0.52) | 0.21 (0.16) | |
| ctx-lh-entorhinal | -0.09 (0.54) | 0.08 (0.60) | ctx-rh-entorhinal | 0.05 (0.74) | 0.27 (0.07) | |
| ctx-lh-parahippocampal | 0.15 (0.34) | 0.19 (0.21) | ctx-rh-parahippocampal | 0.27 (0.08) | 0.28 (0.06) | |
| Left-Hippocampus | 0.19 (0.21) | 0.22 (0.15) | Right-Hippocampus | 0.04 (0.79) | 0.17 (0.25) | |
| Left-Amygdala | 0.23 (0.12) | 0.04 (0.78) | Right-Amygdala | 0.11 (0.46) | -0.05 (0.75) | |
| ctx-lh-inferiortemporal | -0.19 (0.20) | -0.06 (0.69) | ctx-rh-inferiortemporal | -0.06 (0.71) | -0.11 (0.46) | |
| ctx-lh-fusiform | -0.16 (0.29) | -0.02 (0.88) | ctx-rh-fusiform | -0.19 (0.20) | -0.06 (0.72) | |
| ctx-lh-middletemporal | 0.09 (0.54) | 0.11 (0.46) | ctx-rh-middletemporal | 0.35 (0.02*) | 0.38 (0.01*) | |
| ctx-lh-bankssts | 0.31 (0.04*) | 0.20 (0.19) | ctx-rh-bankssts | 0.21 (0.18) | 0.07 (0.65) | |
| ctx-lh-superiortemporal | 0.18 (0.25) | 0.14 (0.37) | ctx-rh-superiortemporal | 0.10 (0.52) | -0.03 (0.86) | |
| ctx-lh-transversetemporal | 0.12 (0.42) | -0.01 (0.97) | ctx-rh-transversetemporal | -0.05 (0.74) | -0.27 (0.08) | |
| ctx-lh-supramarginal | 0.24 (0.11) | 0.19 (0.21) | ctx-rh-supramarginal | 0.47 (0.001*) | -0.07 (0.63) | |
| ctx-lh-insula | 0.27 (0.08) | 0.32 (0.03*) | ctx-rh-insula | -0.04 (0.81) | 0.14 (0.35) | |
| ctx-lh-rostralanteriorcingulate | 0.07 (0.64) | 0.09 (0.56) | ctx-rh-rostralanteriorcingulate | 0.10 (0.52) | 0.26 (0.08) | |
| ctx-lh-caudalanteriorcingulate | -0.01 (0.96) | 0.10 (0.50) | ctx-rh-caudalanteriorcingulate | 0.09 (0.58) | 0.37 (0.01*) | |
| ctx-lh-posteriorcingulate | 0.04 (0.79) | 0.19 (0.20) | ctx-rh-posteriorcingulate | 0.07 (0.63) | 0.35 (0.02*) | |
| ctx-lh-isthmuscingulate | 0.21 (0.17) | 0.07 (0.67) | ctx-rh-isthmuscingulate | 0.37 (0.01*) | 0.17 (0.26) | |
| ctx-lh-precuneus | 0.19 (0.23) | -0.03 (0.82) | ctx-rh-precuneus | 0.17 (0.25) | 0.06 (0.70) | |
| ctx-lh-paracentral | 0.18 (0.23) | 0.22 (0.14) | ctx-rh-paracentral | 0.06 (0.69) | -0.05 (0.74) | |
| ctx-lh-postcentral | 0.15 (0.33) | 0.13 (0.39) | ctx-rh-postcentral | 0.33 (0.03*) | 0.30 (0.05*) | |
| ctx-lh-precentral | 0.27 (0.07) | 0.22 (0.15) | ctx-rh-precentral | 0.12 (0.44) | 0.17 (0.26) | |
| ctx-lh-superiorparietal | 0.24 (0.12) | -0.13 (0.39) | ctx-rh-superiorparietal | 0.19 (0.21) | -0.24 (0.11) | |
| ctx-lh-inferiorparietal | 0.30 (0.05*) | 0.16 (0.30) | ctx-rh-inferiorparietal | 0.12 (0.45) | 0.34 (0.02*) | |
| ctx-lh-lateraloccipital | -0.16 (0.31) | 0.09 (0.56) | ctx-rh-lateraloccipital | -0.13 (0.41) | 0.25 (0.10) | |
| ctx-lh-cuneus | 0.01 (0.95) | 0.07 (0.67) | ctx-rh-cuneus | -0.08 (0.58) | -0.07 (0.66) | |
| ctx-lh-pericalcarine | -0.01 (0.94) | -0.01 (0.94) | ctx-rh-pericalcarine | 0.01 (0.95) | 0.01 (0.94) | |
| ctx-lh-lingual | 0.22 (0.14) | 0.11 (0.47) | ctx-rh-lingual | 0.07 (0.66) | 0.08 (0.62) | |
| Left-Thalamus-Proper | 0.39 (0.01*) | 0.23 (0.13) | Right-Thalamus-Proper | 0.19 (0.23) | 0.24 (0.11) | |
| Left-Caudate | 0.09 (0.58) | 0.29 (0.05*) | Right-Caudate | 0.11 (0.49) | 0.23 (0.12) | |
| Left-Putamen | 0.11 (0.48) | 0.07 (0.64) | Right-Putamen | 0.22 (0.14) | 0.24 (0.11) | |
| Left-Pallidum | 0.22 (0.16) | 0.29 (0.05*) | Right-Pallidum | 0.19 (0.22) | 0.25 (0.09) | |
| Left-Accumbens-area | 0.40 (0.01*) | 0.40 (0.01*) | Right-Accumbens-area | 0.21 (0.16) | 0.15 (0.34) | |
| Left-VentralDC | 0.06 (0.70) | 0.09 (0.56) | Right-VentralDC | 0.00 (0.98) | 0.02 (0.91) | |

Data are Pearson *r* (*P* values) for correlations between bilateral Ch1-3 and Ch4 volumes and the volume of each lateralized FreeSurfer [Version 6.0] ROI from the Desikan-Killiany atlas. Degrees of freedom for Pearson correlation coefficients is 43. * *P* < 0.05. Abbreviations: *Ch1-3*, cholinergic nuclei 1-3; *Ch4*, cholinergic nuclei.

**Supplementary Figure 1.**

Mean global cortical amyloid burden by *APOE* genotype without (A) and with (B) partial volume correction. Group differences in global cortical amyloid burden by *APOE* genotype were calculated using one-way ANOVA and pairwise comparisons were conducted using t-tests with Bonferroni adjustment. *F* statistics and *P* values for each model, along with Jockheere-Terpstra statistics for the assessment of monotonic trends and its statistical significance, are depicted below the x-axis and t-tests and *P* values of pairwise comparisons are depicted above the bar graphs to denote significant group differences. Global cortical Aβ burden was calculated as a weighted average of composite regions commonly affected by Aβ burden in AD, which included prefrontal, lateral temporal, posterior cingulate/precuneus, and lateral parietal ROIs. Abbreviations: *Aβ*, amyloid beta; *AD*, Alzheimer’s disease; *ANOVA*, Analysis of Variance; *APOE*, apolipoprotein E; *PiB*; Pittsburg compound B; *IY-PVC*, Iterative Yang partial volume correction; *DVR*, distribution volume ratio.

**Supplementary Figure 2.**

Amyloid burden in basal forebrain substructures by *APOE* genotype after partial volume correction. Group differences in BFCS amyloid burden by *APOE* genotype were calculated using one-way ANOVA and pairwise comparisons were conducted using t-tests with Bonferroni adjustment. *F* statistics and *P* values for each model are depicted below the x-axis and t-tests and *P* values of pairwise comparisons are depicted above the bar graphs to denote significant group differences. Abbreviations: *BFCS*, basal forebrain cholinergic system; *PiB*; Pittsburg compound B; *IY-PVC*, Iterative Yang partial volume correction; *DVR*, distribution volume ratio; *Ch1-3*, cholinergic nuclei 1-3; *Ch4*, cholinergic nuclei 4.

**Supplementary Figure 3.**

Association between partial volume-corrected mean global cortical amyloid burden and (A) Ch1-3 volume and (B) Ch4 volume. Displayed are Pearson *r* (*P*) for correlations between untransformed and log-transformed global cortical amyloid burden *(DVR)* with basal forebrain volumes. P-values for associations with log-transformed global cortical amyloid burden have been derived using one-sided permutation tests. Global cortical Aβ burden was calculated as a weighted average of composite regions commonly affected by Aβ burden in AD, which included prefrontal, lateral temporal, posterior cingulate/precuneus, and lateral parietal ROIs. Degrees of freedom for Pearson correlation coefficients with untransformed amyloid burden is 43. Abbreviations: *Aβ*, amyloid beta; *AD*, Alzheimer’s disease; *IY-PVC*, Iterative Yang partial volume correction; *PiB*; Pittsburg compound B; *DVR*, distribution volume ratio; *Ch1-3*, cholinergic nuclei 1-3; *Ch4*, cholinergic nuclei 4.

**Supplementary Figure 4.**

Association between mean global cortical amyloid burden and (A) Ch1-3 volume and (B) Ch4 volume in Aβ + and Aβ- participants using a [^11^C]PiB *DVR* threshold of 1.2. Shown are Pearson *r* (*P*) for correlations between untransformed and log-transformed mean global cortical amyloid burden (*DVR*) with basal forebrain volumes, with separate models for Aβ + and Aβ- participants. P-values for associations with log-transformed global cortical amyloid burden have been derived using one-sided permutation tests. A *DVR* threshold of 1.2 was used to determine amyloid status (positive vs. negative). Global cortical Aβ burden was calculated as a weighted average of composite regions commonly affected by Aβ burden in AD, which included prefrontal, lateral temporal, posterior cingulate/precuneus, and lateral parietal ROIs. Degrees of freedom for Pearson correlation coefficients with untransformed amyloid burden is 43. Abbreviations: *Aβ*, amyloid beta; *AD*, Alzheimer’s disease; *PiB*; Pittsburg compound B; *DVR*, distribution volume ratio; *Ch1-3*, cholinergic nuclei 1-3; *Ch4*, cholinergic nuclei 4.

**Supplementary Figure 5.**

Group differences in BFCS volume by amyloid status using a mean global cortical amyloid *DVR* threshold of 1.2. Bar graphs represent mean (SD) for Ch1-3 and Ch4 basal forebrain volumes (cm^3^) for amyloid positive and negative participants. Volumes were normalized using each participant’s eICV. T-tests and *P* values of pairwise comparisons are depicted above the bar graphs to denote significant group differences. A mean global cortical [^11^C]PiB *DVR* threshold of 1.2 was used to determine amyloid status (positive vs. negative). Non-partial volume corrected global cortical Aβ burden was calculated as a weighted average of composite regions commonly affected by Aβ burden in AD, which included prefrontal, lateral temporal, posterior cingulate/precuneus, and lateral parietal ROIs. Abbreviations: *Aβ*, amyloid beta; *AD*, Alzheimer’s disease; *PiB*; Pittsburg compound B; *DVR*, distribution volume ratio; *eICV*, estimated intracranial volume; *Ch1-3*, cholinergic nuclei 1-3; *Ch4*, cholinergic nuclei 4.

**Supplementary Figure 6.**

Association between mean global cortical amyloid burden and (A) Ch1-3 volume and (B) Ch4 volume in Aβ + and Aβ- participants using a [^11^C]PiB *DVR* threshold of 1.08. Data are Pearson *r* (*P*) for correlations between untransformed and log-transformed mean global cortical amyloid burden (*DVR*) and basal forebrain volumes, with separate models for Aβ + and Aβ- participants. P-values for associations with log-transformed global cortical amyloid burden have been derived using one-sided permutation tests. A *DVR* threshold of 1.08 was used to determine amyloid status (positive vs. negative). Global cortical Aβ burden was calculated as a weighted average of composite regions commonly affected by Aβ burden in AD, which included prefrontal, lateral temporal, posterior cingulate/precuneus, and lateral parietal ROIs. Degrees of freedom for Pearson correlation coefficients with untransformed amyloid burden is 43. Abbreviations: *Aβ*, amyloid beta; *AD*, Alzheimer’s disease; *PiB*; Pittsburg compound B; *DVR*, distribution volume ratio; *Ch1-3*, cholinergic nuclei 1-3; *Ch4*, cholinergic nuclei 4.

**Supplementary Figure 7.**

Association between partial volume-corrected mean global cortical amyloid burden and (A) hippocampal, (B) parahippocampal, (C) entorhinal, and (D) amygdalar volume. Displayed are Pearson *r* (*P*) for correlations between untransformed and log-transformed global cortical amyloid burden *(DVR)* and medial temporal lobe volumes. P-values for associations with log-transformed global cortical amyloid burden have been derived using one-sided permutation tests. Global cortical Aβ burden was calculated as a weighted average of composite regions commonly affected by Aβ burden in AD, which included prefrontal, lateral temporal, posterior cingulate/precuneus, and lateral parietal ROIs. Degrees of freedom for Pearson correlation coefficients with untransformed amyloid burden is 43. Abbreviations: *Aβ*, amyloid beta; *AD*, Alzheimer’s disease; *IY-PVC*, Iterative Yang partial volume correction; *PiB*; Pittsburg compound B; *DVR*, distribution volume ratio.

**Supplementary Figure 8.**

Association between mean global cortical amyloid burden and (A) hippocampal, (B) parahippocampal, (C) entorhinal, and (D) amygdalar volume in Aβ + and Aβ- participants using a [^11^C]PiB *DVR* threshold of 1.2. Data are Pearson *r* (*P*) for correlations between untransformed and log-transformed mean global cortical amyloid burden (*DVR*) with medial temporal lobe volumes, with separate models for Aβ + and Aβ- participants. P-values for associations with log-transformed global cortical amyloid burden have been derived using one-sided permutation tests. A *DVR* threshold of 1.2 was used to determine amyloid status (positive vs. negative). Global cortical Aβ burden was calculated as a weighted average of composite regions commonly affected by Aβ burden in AD, which included prefrontal, lateral temporal, posterior cingulate/precuneus, and lateral parietal ROIs. Degrees of freedom for Pearson correlation coefficients with untransformed amyloid burden is 43. Abbreviations: *Aβ*, amyloid beta; *AD*, Alzheimer’s disease; *PiB*; Pittsburg compound B; *DVR*, distribution volume ratio.

**Supplementary Figure 9.**

Group differences in medial temporal lobe volume by amyloid status using a mean global cortical amyloid *DVR* threshold of 1.2. Bar graphs represent mean (SD) for medial temporal lobe volumes (cm^3^) for amyloid positive and negative participants. Volumes were normalized using each participant’s eICV. Aβ+ and Aβ- groups, defined based on the mean global cortical [^11^C]PiB *DVR* threshold of 1.2, did not show group differences in volumes determined using t-tests (degree of freedom=43). Global cortical Aβ burden was calculated as a weighted average of composite regions commonly affected by Aβ burden in AD, which included prefrontal, lateral temporal, posterior cingulate/precuneus, and lateral parietal ROIs. Abbreviations: *Aβ*, amyloid beta; *AD*, Alzheimer’s disease; *PiB*; Pittsburg compound B; *DVR*, distribution volume ratio; *eICV*, estimated intracranial volume.

**Supplementary Figure 10.**

Association between mean untransformed and log-transformed global cortical amyloid burden and (A) hippocampal, (B) parahippocampal, (C) entorhinal, and (D) amygdalar volume in Aβ+ and Aβ- participants using a [^11^C]PiB *DVR* threshold of 1.08. Data are Pearson *r* (*P*) for correlations between mean global cortical amyloid burden (*DVR*) and medial temporal lobe volumes, with separate models for Aβ + and Aβ- participants. P-values for associations with log-transformed global cortical amyloid burden have been derived using one-sided permutation tests. A *DVR* threshold of 1.08 was used to determine amyloid status (positive vs. negative). Global cortical Aβ burden was calculated as a weighted average of composite regions commonly affected by Aβ burden in AD, which included prefrontal, lateral temporal, posterior cingulate/precuneus, and lateral parietal ROIs. Degrees of freedom for Pearson correlation coefficients with untransformed amyloid burden is 43. Abbreviations: *Aβ*, amyloid beta; *AD*, Alzheimer’s disease; *PiB*; Pittsburg compound B; *DVR*, distribution volume ratio.
